# Disinfection Efficacy of Laser Activation on Different Forms and Concentrations of Sodium Hypochlorite Root Canal Irrigant against *Enterococcus faecalis* in Primary Teeth

**DOI:** 10.3390/children10121887

**Published:** 2023-12-03

**Authors:** Chandrashekar Murugesh Yavagal, Srinivas K. Subramani, Viplavi Chavan Patil, Puja C. Yavagal, Ramachandra P. Talwar, Mamata Iranna Hebbal, Selma A. Saadaldin, Elzahraa Eldwakhly, Manal M. Abdelhafeez, Mai Soliman

**Affiliations:** 1 Department of Pediatric Dentistry, Maratha Mandal’s Nathajirao G. Halgekar Institute of Dental Sciences & Research Centre, Belgaum 590019, Karnataka, India; 2 Department of Public Health Dentistry, Bapuji Dental College and Hospital, Davanagere 577004, Karnataka, India; 3 Department of Preventive Dental Sciences, College of Dentistry, Princess Nourah Bint Abdulrahman University, P.O. Box 84428, Riyadh 11671, Saudi Arabia; 4 Prosthodontics Division, Schulich School of Medicine and Dentistry, Western University, London, ON N6A 3K7, Canada; 5 Department of Clinical Dental Sciences, College of Dentistry, Princess Nourah Bint Abdulrahman University, P.O. Box 84428, Riyadh 11671, Saudi Arabia; 6 Department of Conservative Dental Sciences, College of Dentistry, Qassim University, P.O. Box 6666, Buraydah 51452, Saudi Arabia; 7 Department of Endodontics, Faculty of Dentistry, October University for Modern Sciences and Arts, 6th of October City 12451, Egypt

**Keywords:** disinfections, *E. faecalis*, laser, primary teeth, root canal irrigant, sodium hypochlorite

## Abstract

Photoactivated disinfection with sodium hypochlorite (NaOCl) has improved primary root canal treatment outcomes. This in vitro study aims to assess and compare the disinfecting efficacy of 2.5% sodium hypochlorite solution and 5.25% sodium hypochlorite gel, without laser activation and accompanied by laser activation, on *Enterococcus faecalis*-contaminated primary teeth root canals. After one month of incubating extracted teeth specimens with *E. faecalis*, 36 specimens were randomly divided into two groups: Group A (conventional method without laser-activated irrigation) and Group B (with laser-activated irrigation). Each group was further divided into three subgroups, with six samples in each subgroup. Subgroup 1 received irrigation with normal saline, Subgroup 2 with 2.5% sodium hypochlorite solution, and Subgroup 3 with 5.25% sodium hypochlorite gel. Diode laser activation at 810 nm was used in Group B. Bacterial colony counts were measured before and after the intervention. Student’s *t*-test and one-way analysis of variance (ANOVA) with Tukey’s post hoc test were used for statistical analysis. The significance level was set at *p* < 0.05. Microbial analysis revealed no bacterial growth in samples irrigated with 5.25% sodium hypochlorite gel activated with the laser. Activation with the laser significantly (*p* = 0.02) improved the disinfection ability of the irrigant compared to the non-activation group. The disinfection ability of sodium hypochlorite gel was better than that of saline (*p* = 0.02); however, it was comparable to that of sodium hypochlorite solution (*p* = 0.67). Conclusion: Root canal irrigation with 5.25% sodium hypochlorite gel activated with an 810 nm diode laser resulted in complete eradication of *Enterococcus faecalis*, indicating its effectiveness as an endodontic disinfection treatment modality.

## 1. Introduction

Effective debridement and disinfection of the root canal space are crucial for the success of primary teeth endodontic treatment [1]. The main objective of root canal treatment is to eliminate microorganisms and their by-products from the root canal and prevent their re-entry [2]. Mechanical debridement can reduce bacterial counts but cannot completely eradicate them. To further decrease intra-radicular bacteria, various root canal irrigants and disinfection techniques are necessary. Moreover, there is a deficiency of evidence for complete microbial elimination [3].

The complex morphology of primary teeth root canals and the presence of bacteria in biofilms contribute to the incomplete eradication of intra-radicular bacteria, thereby hindering the antimicrobial activity of root canal irrigants [3]. The biofilms found in deciduous teeth with necrotic pulps mainly consist of anaerobic microorganisms, with aerobic microorganisms present in 60% of cases, streptococci in 85%, and Gram-negative bacilli in 15% of cases [4]. Gram-positive bacteria, for instance, Streptococcus and *Enterococcus faecalis*, are responsible for most endodontic failures due to their resistance to conventional treatment.

*Enterococcus faecalis*, a Gram-positive anaerobic coccus, can survive harsh conditions and withstand inadequate nutrition, with the capability of forming complex biofilms [3]. *E. faecalis*-dominant biofilms are highly resistant to conventional irrigants, which is attributed to the formation of extracellular polymeric matrix. Moreover, biofilms provide nutrition and protection to bacteria, increasing their resistance to antimicrobial agents [3].

Sodium hypochlorite is the preferred root canal irrigation material used in endodontic treatment due to its superior antibacterial properties, low surface tension, ease of use, and affordability [4]. However, its optimum used concentration, contact time, and temperature for clinical use are still debated. In paediatric dentistry, a concentration of 2.5% NaOCl is recommended to prevent complications related to pathological resorption areas in primary teeth roots. Although NaOCl effectively disinfects the root canal and dissolves soft tissue and pulpal residuals, its cytotoxicity can cause an acute injury to the periapical area if extruded beyond the apex [5]. The extrusion of NaOCl beyond the apex has been reported in about 42% of endodontic practitioners’ careers, with factors such as high syringe pressure, needle wedging, and large apex size playing a role, particularly in immature teeth [5]. The use of NaOCl in gel form has been introduced to minimise the potential side-effects associated with liquid NaOCl [6]. Studies have shown that the gel form reported significantly less extrusion in comparison with the solution form, especially when the apex diameter was less than 2.5 mm [4]. Sodium hypochlorite gel is suggested as a safer alternative with similar antimicrobial action as the NaOCl solution but with reduced apical extrusion.

Laser-photoactivated disinfection when combined with sodium hypochlorite offers several benefits, including fast penetration of the medication into the root canal, complete penetration of photosensitisers into biofilms and dentinal tubules, limited penetration and cytotoxicity in adjoining tissues, and absence of thermal side-effects [7]. The combination of a diode laser (940 nm) and NaOCl has a synergistic effect, enhancing the bactericidal action [8]. However, there is a lack of literature on the combination effects of sodium hypochlorite in gel form activated by an 810 nm diode laser for endodontic disinfection of deciduous root canals. Hence, an in vitro study was planned to test the null hypothesis, stating that there was no difference in the disinfecting efficacy of 2.5% sodium hypochlorite solution and 5.25% sodium hypochlorite gel against *Enterococcus faecalis*-contaminated primary teeth root canals, without laser activation and accompanied by 810 nm diode laser activation.

## 2. Materials and Methods

This in vitro study was granted ethical approval by the Institutional Review Board, Maratha Mandal’s, Nathajirao G. Halgekar Institute of Dental, Sciences & Research Centre, Belagavi, with number (2020-20/1391).

### 2.1. Study Design (Figure 1)

#### 2.1.1. Inclusion and Exclusion Criteria

The extracted primary teeth specimens were collected from the Department of Pedodontics and Preventive dentistry after obtaining consent from the donors, and then stored in normal saline. The inclusion criteria of the study samples were set based on the study conducted by Yavagal et al. [9].

Inclusion criteria involved the extracted primary teeth with:

- At least two thirds of root length developed.
- Single-rooted and multi-rooted teeth.
- Teeth extracted for therapeutic reasons (over-retained primary teeth, teeth causing ectopic eruption, etc.).
- Teeth with no internal or external pathological root resorption.
- Teeth with no internal or external perforation in the furcation area.

Exclusion criteria involved extracted primary teeth with:

- Root resorption extending beyond half of the total root length.
- Obliterated root canals.

**Figure 1:**
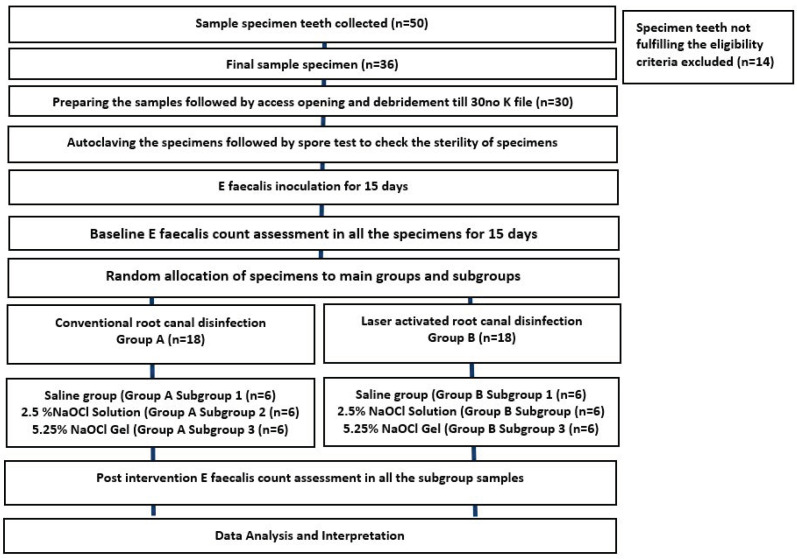
Schematic diagram of the methodology.

#### 2.1.2. Preparation of Teeth Specimen

After collection of extracted deciduous teeth fulfilling the eligibility criteria, debris, calculus, and soft tissue remains around the root surfaces were cleaned using a Gracey curette and all the teeth were placed in saline solution until further use. To obtain standardised tooth lengths, the teeth were decoronated to be of 10 mm in length using a diamond disc. The canal patency was verified using #10 K-file (Mani, Tochigi, Japan) and the working length was maintained at 9 mm. Root canals were prepared using Manual K file Mani, Japan, until size 25. Root canals were rinsed with 2 mL of 2.5% sodium hypochlorite (Vensons India, Bangalore) in between the filing procedures.

After instrumentation, the canals were rinsed with 1 mL of 17% Ethylenediamine tetraacetic acid (EDTA), 5 mL saline, and 1 mL of sodium hypochlorite for 3 min each to remove the smear layer. Then, as a final flush, all the canals were rinsed with 5 mL saline. The apical foramen was sealed with self-cure glass ionomer cement (GC Gold Label 2 Glass Ionomer Restorative cement) and the root surfaces were covered with two layers of nail varnish. Each tooth specimen was transferred to a 2 mL centrifuge tube and then autoclaved at 121 °C under a pressure of 15 psi for 30 min.

#### 2.1.3. Bacterial Inoculation

Pure culture of *E. faecalis* American Type Culture Collection (ATCC^®^ 29212™) was left to grow overnight in brain heart infusion broth (M210-HiMedia Laboratories Pvt. Limited, Maharashtra, India), and a bacterial inoculum suspension was created. McFarland barium sulfate (BaSO_4_) standard (approximately 10^8^ colony-forming units per milliliter (CFUs/mL)) was used in measuring the optical density and calibrating the values using spectrophotometry at wavelength 600 nm. The prepared bacterial inoculum turbidity was standardized against 0.5 McFarland standard. The root canals were then filled with 30 µL of *E. faecalis* suspension by centrifugation at 10,000× *g* for 5 min and then suspended in saline and adjusted to 3 × 10^6^ cells/mL using a spectrophotometer. All specimens were kept immersed in broth at 37 °C to allow bacterial growth and the medium was replaced once a week for 4 successive weeks. Incubatory growth was confirmed in the canals of specimens by taking the inoculums of the canals and checking *E. faecalis* colony count on culture plates. The methodology followed for bacterial inoculation of canals was based on the study conducted by Yavagal et al. [9].

#### 2.1.4. Sample Size Calculation

Sample size was calculated using G Power sample size estimation software (Version 3.1.9.7). Power analysis was carried out for a 3 × 2 fixed-effects analysis of variance; the first factor (non-activated irrigants) and second factor (laser-activated irrigants) included three sublevels each, creating 6 subgroups. Minimum expected difference was derived from the study conducted by Roshdy et al., 2018 [3]. Fixing alpha at 0.05 and power at 0.8, the estimated sample size was 36 specimens with 6 specimens within each subgroup.

#### 2.1.5. Randomisation

Thirty-six teeth specimens which were prepared and inoculated with *E. faecalis* were coded and allocated randomly to 6 subgroups. Random number sequence was generated using online software. Allocation of specimens to interventional groups was concealed.

#### 2.1.6. Interventional Groups

In Group A (control group), root canal irrigation and disinfection were carried out using the conventional method without laser activation. In Group B (experimental group), laser-activated root canal irrigation and disinfection were carried out. Each group comprised three subgroups based on the irrigation agent used.

Subgroup 1: irrigation of root canals with 5 mL of saline for 5 min.

Subgroup 2: irrigation of root canals with 5 mL of 2.5% sodium hypochlorite for 5 min.

**Figure 2:**
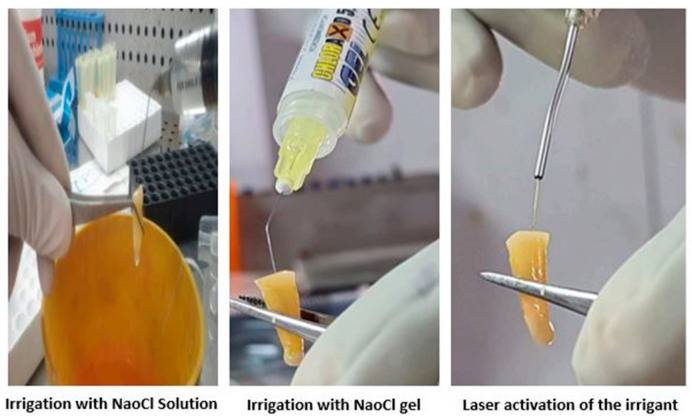
Interventional groups.

Subgroup 3: Irrigation of root canals with 5 mL of CHLORAXID 5.25% Gel (Sun Dental and Medical Devices, Mumbai India for 5 min following the manufacturer’s instructions. Irrigation was carried out using a 30 G needle, inserted 1 mm shorter than the working length (Figure 2).

### 2.2. Baseline Microbial Analysis

At baseline, a microbial sample was obtained from each canal immediately before disinfection of root canal and inoculated on brain heart infusion media, and then incubated for 24–48 h to check for the growth of *E. faecalis*.

### 2.3. Diode Laser Activation of Irrigants

In Group B (experimental group), after irrigation of the root canals, laser activation was performed in each subgroup with a special endodontic diode laser with a wavelength of 810 nm (Novolase Dual wave Endo Diode, Novolase Technologies, India) at an output power of 1 watt in continuous wave mode. Laser activation was carried out three times for 15 s each, with a 5 s interval between irradiations. The laser activation was delivered to the canal until it was 1 mm shorter than the working length using a special endodontic fibre tip, 200 µm in diameter (Novolase Technologies, Bengaluru, India). The hand piece was grasped at around an angle of 10 degrees between the fibre and the root canal wall. Irradiation was performed by circular movements starting at the apical part towards the coronal part (step-back technique) of the root canal. The procedure was carried out by a certified laser specialist who was blinded to the irrigants used.

### 2.4. Post-Intervention Microbial Analysis

Root canals were filled with sterile saline solution using a 30 G syringe, and dentin was scraped from inside the canals using a Hedstrom file #25. Then, a sterile paper point #25 was used to dry the canals for 60 s and then immersed in thioglycolates broth in 1.5 mL Eppendorf tubes, before being vortexed for 30 s and incubated for 24–48 h at 37 °C. Then, the inoculated broth suspension samples were inoculated in BHI agar and incubated for 48 h at 37 °C in a CO_2_ jar. After incubation, the colony-forming units of *E. faecalis* were assessed. All previous steps were performed under sterile aseptic conditions by a microbiologist who was blinded to interventional details.

### 2.5. Statistical Analysis

Statistical package for social sciences (SPSS) version 22 (IBM SPSS Statistics for Windows, Version 22.0. Armonk, NY, USA: IBM Corp) was used for statistical analysis. Data followed normal distribution. Mann–Whitney U Tests were used for group comparison and the Wilcoxon signed rank test was used for within-group comparisons between two groups. One-way analysis of variance (Kruskal–Wallis ANOVA) followed by the post hoc Mann–Whitney Test was used to compare subgroups. The significance level was set at *p* < 0.05.

## 3. Results

The pre-intervention bacterial colony-forming unit (CFU) values were in the range of 10^7^, whereas post intervention, the values ranged from 0 to 10^6^. The distribution was skewed; hence, a log10 transformation of data was performed and is presented.

In the current study, no statistically significant difference was reported in the *E. faecalis* colony-forming units (CFUs) between the subgroups both in the control and experimental groups pre-intervention. However, a statistically significant reduction in the *E. faecalis* CFUs post intervention in both groups was reported. The post hoc test revealed that the disinfection ability of sodium hypochlorite solution and gel was better than that of saline (*p* = 0.03, *p* = 0.004, respectively); however, there was no significant difference between sodium hypochlorite gel and solution. Similar results were observed in experimental groups. Sodium hypochlorite solution and gel were better than saline (*p* = 0.09, *p* = 0.02) (Table 1).

**Figure 3:**
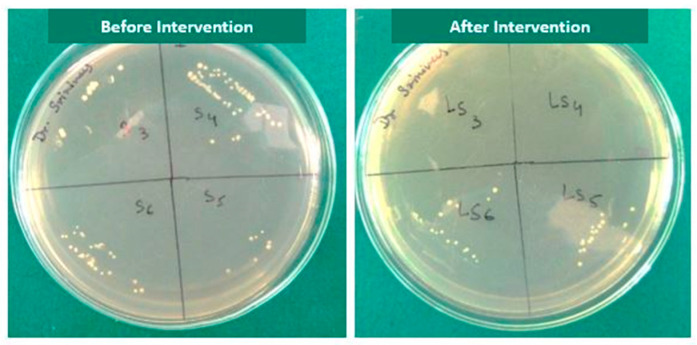
Culture media showing growth of *E. faecalis* before and after laser activated irrigation in all the subgroups.

A subgroup comparison between the control and experimental group revealed that there was no statistically significant difference except for saline (0.004) and sodium hypochlorite gel post intervention (0.02). There was a marked reduction in the *E. faecalis* counts post intervention in all the subgroups with a maximum reduction in the NaOCl gel group. In general, laser activation significantly improved the disinfection ability of the irrigant compared to non-activation (Table 2, Figure 3).

## 4. Discussion

The study results favor acceptance of the null hypothesis where there was no significant difference between the disinfecting efficacy of 2.5% sodium hypochlorite solution and 5.25% sodium hypochlorite gel against *Enterococcus faecalis*-contaminated primary teeth root canals without laser activation. However, laser activation of sodium hypochlorite gel showed a superior disinfecting efficacy compared to non-activated gel.

A comparable result was reported in an in vivo study performed by Kotecha et al., where they treated multirooted primary teeth that had endodontic lesions using 5.25% sodium hypochlorite gel and solution, with both the interventions demonstrating comparable antimicrobial effectiveness when implemented as root canal disinfectants [10]. In addition, other in vitro studies have also demonstrated comparable efficacy of both sodium hypochlorite gel and solution [11,12,13].

Other studies by Hasna et al. and Luz et al. concluded that even though NaOCl solution reported a higher capacity for tissue dissolution than that of the gel form, both the sodium gel and solution forms were efficient in reducing the microbial load of *E. faecalis* [11,12].

Contrasting results were observed in a study by Zand et al. where sodium hypochlorite in solution form exhibited better antibacterial efficacy than the gel form [14], while a clinical study by Karatas et al. using NaOCl gel during root canal preparation resulted in reduced post-operative pain on day 1 when compared to NaOCl solution [15].

However, an in vitro investigation by Faria et al. demonstrated that the 3% sodium hypochlorite solution reported better penetrability in dentinal tubules (*p* < 0.05) than the gel form, and these results indicated that the gel form penetration into dentinal tubules is limited due to the increase in viscosity. This can be considered a limitation of the NaOCl gel form, in addition to other problems like the dubious penetration into unreachable regions, the inability to remove debris from root canals, and needing to add saline solution in addition to the gel for passive ultrasonic irrigation, which could dilute and lose the effectiveness of NaOCl gel [16].

In the current study, root canal irrigation with 5.25% NaOCl gel activated with an 810 nm diode laser resulted in the complete eradication of *Enterococcus faecalis*. However, a few studies showed the bactericidal efficacy of a 940 nm diode-laser-activated 5% NaOCl solution used as an irrigant against *E. faecalis* [8,17,18,19,20]. Moreover, the bactericidal efficiency improved by using the erbium-laser-activated irrigation (LAI) of 0.5% NaOCl against *E. faecalis* biofilm in a few studies [21,22,23].

However, Cristo et al. showed that there is no significant improvement in the antibacterial efficacy of low-powered (0.5 W) Er, Cr:YSGG laser activation on low concentrations of NaOCl used as a root canal irrigant [24].

The photoactivated disinfection mechanism has a significant role in the antibacterial effect of the laser, as when it is combined with NaOCl, it resulted in several benefits; for instance, the acceleration of irrigant penetration inside the root canal, which leads to a rapid bactericidal effect; the complete removal of biofilms and, consequently, the efficient penetration of photosensitisers inside dentinal tubules; and the controlled penetration of the photosensitiser into the adjoining bone and periodontal ligament, which decreases the cytotoxicity and limits the thermal side-effects on the adjacent tissues [25].

As primary teeth exhibit bizarre internal geometry and other features such as furcal connections and horizontal anastomoses that are not common for permanent teeth, the root canal treatment of primary teeth is a more complicated procedure than for permanent teeth [25]. Recent radiographic imaging systems have revealed that remanent pulp tissues might persist due to using mechanical root canal instrumentation only. Hence, irrigation as well as instrumentation together ensure the complete debridement and disinfection of root canals [25].

Furthermore, it was reported that the largest number of microorganisms is present in the main root canal of primary teeth; yet, a significant portion of microorganisms is present in lateral canals, apical ramifications, as well as dentinal tubules. These place a burden on the clinician to accomplish all the aforementioned tasks in a duration that matches the attention span of our young paediatric patients [10].

A mixture of bacterial species is associated with root canal infections in primary teeth, including aerobic and anaerobic microorganisms as well as facultative microorganisms. It was found that *Enterococcus faecalis*, Porphyromonas gingivalis, and Treponema denticola are the most dominant bacterial species found in primary teeth root canals [26]. Also, *E. faecalis* had a reported prevalence of 63% in the necrotic pulp of primary root canals, in addition to being occasionally present in the initial infections of permanent teeth [15].

When NaOCl is added into water, it dissociates into Na^+^ and OCl^−^ ions that form the predominant form of HOCl in acidic or neutral pH media, which are responsible for the antimicrobial effect of the most widely used root canal irrigant [27]. NaOCl is the only irrigant that can dissolve necrotic pulp tissues with a minimum remanent of dentinal collagen; yet, it could not dissolve the smear layer.

However, NaOCl reported cytotoxic activities that may cause acute injuries to the periapical tissues when it extends beyond the root apex, causing haemolysis, ulcerations, and the destruction of endothelial and fibroblast cells, and this resulted in emphysema, trismus, and sensory-motor defects [28]. The extruded irrigant amount might be interrelated to many factors, such as extra pressure from the syringes, needle wedging, and the width of the apical foramen that is noticed frequently in immature teeth [22]. The use of NaOCl in solution form increased the risk of its extrusion beyond the apex, posing a risk to paediatric patients [29].

Some studies found significantly less extrusion of NaOCl in gel form when compared to the solution form, when the apical foramen diameter was less than 2.5 mm. This occurred in spite of the high plunger pressure of the syringe when the gel form was used in comparison to the solution form at the same flow rate [29,30]. Hence, it was decided to test the photo-activated disinfection ability of sodium hypochlorite gel in the present study. Several research studies have found that laser activation leads to a clear modulation in the NaOCl reaction rate that significantly improves the production and consumption of available chlorine and oxygen ions compared to ultrasound activation [31].

The diode laser bactericidal effect could have resulted from its greater penetration depth (1000 μm) in comparison to NaOCl, which penetrates only to 100 μm of the dentinal tubules [21]. Also, diode laser thermal photodisruptive action at unreachable parts of dentin resulted in an improvement in its bactericidal action [32].

In the current study, a fine diameter of the fibre-optic tip of 200 µm was used to improve the delivery of laser light to the root canal walls efficiently, resulting in a reduced bacterial count. Jambagi et al. found that the small fibre-optic tip enhanced the power density of the tip as well as improved the accessibility to the apical third, resulting in effectively extending its use with curved canals [32].

The study’s strength lies in its preliminary attempt to evaluate the disinfection efficacy of laser-activated sodium hypochlorite gel with the traditional technique. However, further in vivo studies with a large sample size should be conducted to explore and validate the evidence related to the study findings. Disinfection efficacy could be tested on root canal biofilm models rather than on a single microorganism. Further studies testing different wavelengths and doses of lasers and the disinfection efficacy of photo activated therapy could give valuable insights into endodontic disinfection therapy.

## 5. Conclusions

Within the limitation of the current in vitro setting, the utilisation of 5.25% sodium hypochlorite gel as a root canal irrigant, activated with an 810 nm diode laser, led to the complete eradication of *Enterococcus faecalis* in primary teeth root canals. If clinical trials confirm the disinfection effectiveness of laser activation of sodium hypochlorite gel, this innovative approach, known for its safety and patient-friendliness, has the potential to significantly enhance the clinical outcomes of root canal therapy in primary teeth, promoting cutting-edge dental care for young patients.

## Figures and Tables

**Table 1:** Comparison of *E. faecalis* counts in the control and interventional groups within the subgroups.

Group		Subgroup	Mean (SD)	Range	Median (Q1–Q3)	Kruskal–Wallis Test	Mann–Whitney U Test		
						*p*-Value	Saline vs. NaOCl Solution	Saline vs. NaOCl Gel	NaOCl Solution vs. NaOCl Gel
Group A (Control Group)	Pre-Intervention	Saline	7.609 (0.046)	7.556–7.663	7.607 (7.565–7.656)	0.22 (NS)	0.07 (NS)	0.47 (NS)	0.47 (NS)
		NaOCl solution	7.54 (0.069)	7.447–7.623	7.544 (7.47–7.607)				
		NaOCl gel	7.575 (0.096)	7.462–7.699	7.578 (7.473–7.665)				
	Post Intervention	Saline	5.276 (0.589)	4.778–6.301	5.05 (4.828–5.8)	0.01 *	0.03 *	0.004 *	0.21 (NS)
		NaOCl solution	0.984 (2.41)	0–5.903	0 (0–1.476)				
		NaOCl gel	2.867 (2.229)	0–4.602	4.151 (0–4.376)				
Group B (Experimental group-Laser group)	Pre-Intervention	Saline	7.593 (0.105)	7.491–7.778	7.556 (7.521–7.677)	0.93 (NS)	1.00 (NS)	0.57 (NS)	1.00 (NS)
		NaOCl solution	7.585 (0.089)	7.477–7.699	7.589 (7.498–7.665)				
		NaOCl gel	7.586 (0.043)	7.544–7.644	7.579 (7.544–7.628)				
	Post Intervention	Saline	2.889 (2.248)	0–4.748	4.19 (0–4.34)	0.03 *	0.09 (NS)	0.02 *	0.14 (NS)
		NaOCl solution	0.717 (1.115)	0–2.301	0 (0–2.075)				
		NaOCl gel	0 (0)	0–0	0 (0–0)				

* *p* < 0.05, statistically significant; *p* > 0.05, non-significant, NS.

**Table 2:** Pre- and post-intervention comparison of *E. faecalis* counts in the control and experimental groups.

Subgroup	Group	Pre-Intervention			Post Intervention			Change		Wilcoxon Signed Rank Test
		Mean (SD)	Range	Median (Q1–Q3)	Mean (SD)	Range	Median (Q1–Q3)	Mean (SD)	%	*p*-Value
Saline	Control Group	7.609 (0.046)	7.556–7.663	7.607 (7.565–7.656)	5.276 (0.589)	4.778–6.301	5.05 (4.828–5.8)	2.333 (0.623)	30.63 (8.06)	0.03 *
	Experimental group (Laser group)	7.593 (0.105)	7.491–7.778	7.556 (7.521–7.677)	2.889 (2.248)	0–4.748	4.19 (0–4.34)	4.704 (2.250)	61.95 (29.65)	0.03 *
Mann–Whitney U Test		*p* = 0.38 (NS)			*p* = 0.004 *					
NaOCl solution	Control Group	7.54 (0.069)	7.447–7.623	7.544 (7.47–7.607)	0.984 (2.41)	0–5.903	0 (0–1.476)	6.556 (2.403)	86.98 (31.89)	0.03 *
	Experimental group (Laser group)	7.585 (0.089)	7.477–7.699	7.589 (7.498–7.665)	0.717 (1.115)	0–2.301	0 (0–2.075)	6.868 (1.110)	90.56 (14.66)	0.03 *
Mann–Whitney U Test		*p* = 0.30 (NS)			*p* = 0.67 (NS)					
NaOCl gel	Control Group	7.575 (0.096)	7.462–7.699	7.578 (7.473–7.665)	2.867 (2.229)	0–4.602	4.151 (0–4.376)	4.707 (2.295)	61.93 (29.62)	0.03 *
	Experimental group (Laser group)	7.586 (0.043)	7.544–7.644	7.579 (7.544–7.628)	0 (0)	0–0	0 (0–0)	7.586 (0.043)	100.00 (0.00)	0.03 *
Mann–Whitney U Test		*p* = 0.87 (NS)			*p* = 0.02 *					

* *p* < 0.05, statistically significant; *p* > 0.05, non-significant, NS.

## Data Availability

The data presented in this study are available on request from the corresponding author.

## References

[1] Walia V., Goswami M., Mishra S., Walia N., Sahay D. (2019). Comparative Evaluation of the Efficacy of Chlorhexidine, Sodium Hypochlorite, the Diode Laser and Saline in Reducing the Microbial Count in Primary Teeth Root Canals–An In Vivo Study. J. Lasers Med. Sci..

[2] Sohrabi K., Sooratgar A., Zolfagharnasab K., Kharazifard M.J., Afkhami F. (2016). Antibacterial activity of diode laser and sodium hypochlorite in *Enterococcus faecalis*-contaminated root canals. Iran. Endod. J..

[3] Roshdy N.N., Kataia E.M., Helmy N.A. (2019). Assessment of antibacterial activity of 2.5% NaOCl, Chitosan nano-particles against *Enterococcus faecalis* contaminating root canals with and without diode laser irradiation: An in vitro study. Acta Odontol. Scand..

[4] Öter B., Topcuoglu N., Tank M.K., Cehreli S.B. (2018). Evaluation of antibacterial efficiency of different root canal disinfection techniques in primary teeth. Photomed. Laser Surg..

[5] Haapasalo H.K., Siren E.K., Waltimo T.M., Orstavik D., Haapasalo M.P. (2000). Inactivation of local root canal medicaments by dentine- an in vitro study. Int. Endod. J..

[6] Nesser S.F.A., Bshara N.G. (2019). Evaluation of the apical extrusion of sodium hypochlorite gel in immature permanent teeth: An in vitro study. Dent. Med. Probl..

[7] de Souza E.B., Cai S., Simionato M.R., Lage-Marques J.L. (2008). High-power diode laser in the disinfection in depth of the root canal dentin. Oral Surg. Oral Med. Oral Pathol. Oral Radiol. Endod..

[8] Castelo-Baz P., Martín-Biedma B., Ruíz-Pinon M., Rivas-Mundina B., Bahillo J., Seoane-Prado R., Perez-Estévez A., Gude F., De Moor R., Varela-Patiño P. (2012). Combined sodium hypochlorite and 940 nm diode laser treatment against mature *E. faecalis* biofilms in-vitro. J. Lasers Med. Sci..

[9] Yavagal C.M., Patil V.C., Yavagal P.C., Kumar N.K., Hariharan M., Mangalekar S.B. (2021). Efficacy of Laser Photoacoustic Streaming in Paediatric Root Canal Disinfection-An Ex-Vivo Study. Contemp. Clin. Dent..

[10] Kotecha N., Shah N.C., Doshi R.J., Kishan K.V., Luke A.M., Shetty K.P., Mustafa M., Pawar A.M. (2023). Microbiological Effectiveness of Sodium Hypochlorite Gel and Aqueous Solution When Implemented for Root Canal Disinfection in Multirooted Teeth: A Randomized Clinical Study. J. Funct. Biomater..

[11] Shamsi P.N., Yeganeh L.A.B., Saberi B.V., Azadeh K.F.P., Kashani T. (2017). Antibacterial effect of sodium hypochlorite gel and solution on *Enterococcus faecalis*. J. Dentomaxillofac. Radiol. Pathol. Surg..

[12] Hasna A.A., Da Silva L.P., Pelegrini F.C., Ferreira C.L.R., de Oliveira L.D., Carvalho C.A.T. (2020). Effect of sodium hypochlorite solution and gel with/without passive ultrasonic irrigation on *Enterococcus faecalis*, Escherichia coli and their endotoxins. F1000Research.

[13] Luz L.B., Santana R., Prates A.W., Froelich J., De Melo T.A., Montagner F., Luisi S.B. (2019). Antimicrobial action, ph, and tissue dissolution capacity of 2.5% sodium hypochlorite gel and solution. J. Health Biol. Sci..

[14] Zand V., Lotfi M., Soroush M.H., Abdollahi A.A., Sadeghi M., Mojadadi A. (2016). Antibacterial Efficacy of Different Concentrations of Sodium Hypochlorite Gel and Solution on *Enterococcus faecalis* Biofilm. Iran. Endod. J..

[15] Karataş E. (2020). Postoperative pain after the use of sodium hypochlorite gel and solution forms: A randomized clinical study. Eur. Endod. J..

[16] Faria G., Viola K.S., Coaguila-Llerena H., Oliveira L.R.A., Leonardo R.T., Aranda-García A.J., Guerreiro-Tanomaru J.M. (2019). Penetration of sodium hypochlorite into root canal dentine: Effect of surfactants, gel form and passive ultrasonic irrigation. Int. Endod. J..

[17] Vaid D., Shah N., Kothari D., Bilgi P. (2017). Additive effect of photoactivated disinfection on the antibacterial activity of QMix 2 in 1 against 6-week *Enterococcus faecalis* biofilms: An in vitro study. J. Conserv. Dent..

[18] Attiguppe P.R., Tewani K.K., Naik S.V., Yavagal C.M., Nadig B. (2017). Comparative Evaluation of Different Modes of Laser Assisted Endodontics in Primary Teeth: An In vitro Study. J. Clin. Diagn. Res..

[19] Dai S., Xiao G., Dong N., Liu F., He S., Guo Q. (2018). Bactericidal effect of a diode laser on *Enterococcus faecalis* in human primary teeth—an in vitro study. BMC Oral. Health.

[20] Mehrvarzfar P., Akhavan H., Rastgarian H., Akhlagi N.M., Soleymanpour R., Ahmadi A. (2011). An in vitro comparative study on the antimicrobial effects of bioglass 45S5 vs. calcium hydroxide on *Enterococcus faecalis*. Iran. Endod. J..

[21] Neelakantan P., Cheng C.Q., Mohanraj R., Sriraman P., Subbarao C., Sharma S. (2015). Antibiofilm activity of three irrigation protocols activated by ultrasonic, diode laser or Er: YAG laser in vitro. Int. Endod. J..

[22] Betancourt P., Merlos A., Sierra J.M., Camps-Font O., Arnabat-Dominguez J., Viñas M. (2019). Effectiveness of low concentration of sodium hypochlorite activated by Er,Cr:YSGG laser against *Enterococcus faecalis* biofilm. Lasers Med. Sci..

[23] Aydin S.A., Taşdemir T., Buruk C.K., Çelik D. (2020). Efficacy of Erbium, Chromium-doped Yttrium, Scandium, Gallium and Garnet Laser-activated Irrigation Compared with Passive Ultrasonic Irrigation, Conventional Irrigation, and Photodynamic Therapy against *Enterococcus faecalis*. J. Contemp. Dent. Pract..

[24] Christo J.E., Zilm P.S., Sullivan T., Cathro P.R. (2016). Efficacy of low concentrations of sodium hypochlorite and low-powered Er,Cr:YSGG laser activated irrigation against an *Enterococcus faecalis* biofilm. Int. Endod. J..

[25] Raducka M., Piszko A., Piszko P.J., Jawor N., Dobrzyński M., Grzebieluch W., Mikulewicz M., Skośkiewicz-Malinowska K. (2023). Narrative Review on Methods of Activating Irrigation Liquids for Root Canal Treatment. Appl. Sci..

[26] Ahmed H.M. (2013). Anatomical challenges, electronic working length determination and current developments in root canal preparation of primary molar teeth. Int. Endod. J..

[27] Wong J., Manoil D., Näsman P., Belibasakis G.N., Neelakantan P. (2021). Microbiological Aspects of Root Canal Infections and Disinfection Strategies: An Update Review on the Current Knowledge and Challenges. Front. Oral. Health.

[28] Dioguardi M., Gioia G.D., Illuzzi G., Laneve E., Cocco A., Troiano G. (2018). Endodontic irrigants: Different methods to improve efficacy and related problems. Eur. J. Dent..

[29] Ozlek E., Neelakantan P., Khan K., Cheung G.S., Rossi-Fedele G. (2021). Debris extrusion during root canal preparation with nickel-titanium instruments using liquid and gel formulations of sodium hypochlorite in vitro. Aust. Endod. J..

[30] Naik R.G., Raviraj G.A., Yavagal C.M., Mandroli P. (2017). Diode Lasers for Pediatric Endodontics: State-of-the-Art. J. Dent. Lasers.

[31] Mishra A., Koul M., Abdullah A., Khan N., Dhawan P., Bhat A. (2022). Comparative Evaluation of Antimicrobial Efficacy of Diode Laser (Continuous Mode), Diode Laser (Pulse Mode), and 5.25% of Sodium Hypochlorite in Disinfection of Root Canal: A Short Study. Int. J. Clin. Pediatr. Dent..

[32] Jambagi N., Kore P., Dhaded N.S. (2021). Comparison of Antimicrobial Efficacy of Diode Laser, Ultrasonic Activated and Conventional Irrigation with 2.5% NaOCl during RCT: An Interventional Study. J. Contemp. Dent. Pract..

